# Metformin Inhibits Migration and Invasion by Suppressing ROS Production and COX2 Expression in MDA-MB-231 Breast Cancer Cells

**DOI:** 10.3390/ijms19113692

**Published:** 2018-11-21

**Authors:** Chandler Schexnayder, Kiera Broussard, Demitrius Onuaguluchi, Anthony Poché, Moamen Ismail, LeFontae McAtee, Shawn Llopis, Amber Keizerweerd, Harris McFerrin, Christopher Williams

**Affiliations:** 1 College of Pharmacy, Xavier University of Louisiana, 1 Drexel Drive, New Orleans, LA 70125, USA; cschexn2@xula.edu (C.S.); kbrouss2@xula.edu (K.B.); donuagul@xula.edu (D.O.); apoche@xula.edu (A.P.); lmcAtee@xula.edu (L.M.); sllopis@xula.edu (S.L.); akeizerw@xula.edu (A.K.); 2 Department of Biology, Xavier University of Louisiana, 1 Drexel Drive, New Orleans, LA 70125, USA; mismail@xula.edu (M.I.); hmcferri@xula.edu (H.M.)

**Keywords:** metformin, ICAM1, metastasis, reactive oxygen species, COX2

## Abstract

Background: Several mechanisms of action have been proposed to explain the apparent antineoplastic functions of metformin, many of which are observed at high concentrations that may not be reflective of achievable tissue concentrations. We propose that metformin at low concentrations functions to inhibit ROS production and inflammatory signaling in breast cancer, thereby reducing metastasis. Methods: Using the highly invasive MDA-MB-231 breast carcinoma model, we ascertained the impact of metformin on cell viability by DNA content analysis and fluorescent dye exclusion. Migration and invasion assays were performed using a modified Boyden chamber assay and metastasis was ascertained using the chorioallantoic membrane (CAM) assay. PGE2 production was measured by Enzyme-Linked Immunosorbent Assay (ELISA). COX2 and ICAM1 levels were determined by flow cytometry immunoassay. Results: Metformin acutely decreased cell viability and caused G2 cell cycle arrest only at high concentrations (10 mM). At 100 µM, however, metformin reduced ICAM1 and COX2 expression, as well as reduced PGE2 production and endogenous mitochondrial ROS production while failing to significantly impact cell viability. Consequently, metformin inhibited migration, invasion in vitro and PGE2-dependent metastasis in CAM assays. Conclusion: At pharmacologically achievable concentrations, metformin does not drastically impact cell viability, but inhibits inflammatory signaling and metastatic progression in breast cancer cells.

## 1. Introduction

Biguanides are a class of orally available drugs discovered in the 1920s to have anti-hyperglycemic activity. Metformin, the only biguanide currently available for clinical use in the US, has long been used as a first line therapy for the treatment of type 2 diabetes mellitus. In recent years, studies have shown that the use of metformin was associated with decreased incidence and mortality of breast cancer when compared to patients using other hypoglycemic treatments [1,2,3]. While early reports of the antineoplastic activity of metformin were primarily based on retrospective observational studies, several prospective studies have recently been published showing a correlation of metformin usage and evidence of antineoplastic activity. Two trials of non-diabetic women with early stage endometrial cancer revealed that metformin treatment was associated with a significant reduction in Ki67 staining or tritiated thymidine uptake, suggesting that metformin causes a substantial decrease in endometrial cell proliferation [4,5]. In the breast, metformin reduced expression of Ki67 in carcinoma cells among a cohort of pre-diabetic patients, providing evidence that metformin may also have anti-neoplastic activities in the breast [6].

Various mechanisms for metformin’s action have been proposed. One model suggests a direct effect of metformin on cancer cell survival. In this model, metformin exposure results in impaired mitochondrial function and reduced cellular ATP/AMP ratio, thereby activating AMP-activated kinase, and resulting in apoptosis [7]. This model is supported by studies in which metformin induces AMPK-dependent apoptosis in vitro and inhibits tumorigenesis in xenograft models of breast cancer. Indeed, studies have shown that excess glucose can support glycolysis, limit the ATP exhaustion caused by inhibition of the mitochondrial metabolism, and thereby counteract the pro-apoptotic effect of metformin [8,9]. However, mM concentrations of metformin are often necessary to induce apoptosis or AMPK activation in vitro, and there are questions as to whether these concentrations are pharmacologically achievable in patients. Indeed, pharmacokinetic experiments in xenograft mouse tumors suggest that metformin concentrations do not rise above 100 μM following 125 mg/kg intraperitoneal injection of the drug [10]. Furthermore, there is little evidence that apoptosis is induced by metformin in breast cancer patient biopsies [11]. The second model is indirect, and suggests that metformin improves hyperinsulinemia, and subsequently withdraws insulin/IGF-1 from dependent cancer cells. Studies have shown that hyperinsulinemia and markers of hyperglycemia were largely resolved in metformin-treated patients with or without cancer [12]. However, the direct causation between metformin-induced changes in insulin signaling and tumor growth has yet to be determined. Furthermore, this model does not explain the impact of metformin outside of the context of hyperinsulinemia, either in vitro or in non-diabetic mouse xenograft systems.

Our previous studies have shown that metformin has a concentration dependent effect on breast cancer cell viability, and that metformin causes alterations in inflammatory signaling in breast cancer cells [13]. Here, we investigated the effects of sub-apoptotic concentrations of metformin on inflammatory signaling in breast cancer cells. In our studies, we utilized the highly invasive MDA-MB-231, an estrogen receptor negative (ER-), HER2-breast cancer cell line, which serves as an in vitro model of triple negative cell lines. At μM concentrations, metformin functions by relieving oxidative stress and subsequent inflammatory signaling through COX2 pathways resulting in decreased migration, invasion, and ultimately metastasis, particularly in highly invasive TNBC cells.

## 2. Results

### 2.1. Metformin Exhibits Concentration Dependent Effects on Cell Cycle Progression

**Figure 1:**
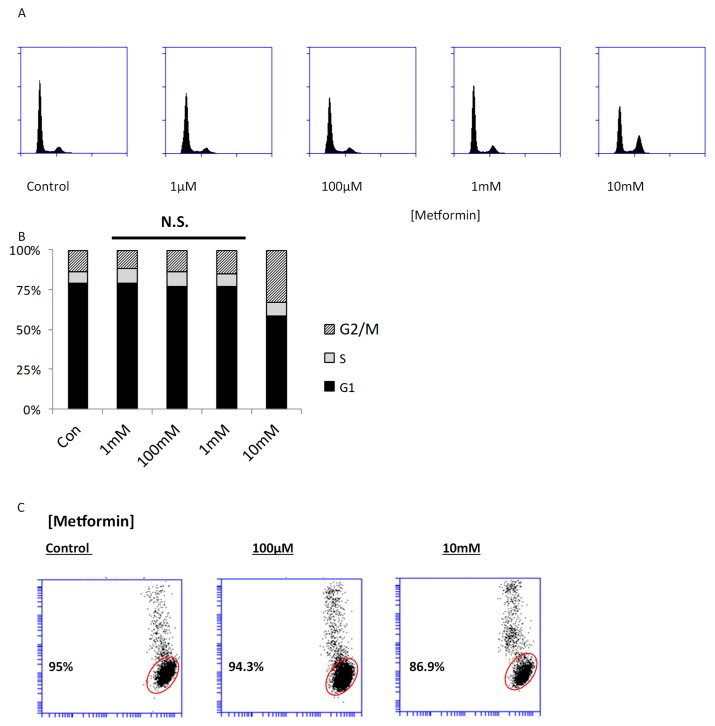
Metformin fails to directly impact breast cancer cell viability at pharmacologically relevant concentrations. (**A**) MDA-MB-231 breast cancer cells were cultured in the presence or absence of metformin for 72 h. Nuclear DNA content was assessed by propidium iodide staining, and analyzed by flow cytometry. The proportion of cells in each phase is depicted in (**B**). In (**C**), cells were cultured in presence or absence of metformin for 48 h, followed by incubation in Sytox Red cell toxicity stain. The dye is excluded from viable cells whose membranes are not compromised (indicated in red). Representative figures are shown. Each assay was performed in quadruplicate. N.S. denotes no significant difference as compared to control.

Several studies have shown that metformin causes apoptosis in breast cancer models; however, the majority of these studies utilized high doses of metformin, typically in the range of 5–20 mM [14,15,16]. Indeed, our previous studies have shown that metformin does not decrease cell viability in breast cancer cells at sub-mM concentrations [13]. To determine the dose-dependent effects of metformin on cell cycle progression, we exposed highly invasive MDA-MB-231 cells to metformin and analyzed DNA content as a measure of cell cycle phase distribution. In control cultures, 13.5% of cells were found in the G2/M phase based on DNA staining intensity (Figure 1A). At concentrations of 1, 100, and 1 mM, 10.6%, 12.8%, and 15.1% of cells were observed in the G2/M phase of the cell cycle. On the other hand, 10 mM metformin resulted in over 30% of cells the G2/M phase, suggesting dose-dependent G2/M arrest. Additionally, metformin failed to significantly induce cell death at any of the concentrations administered, as determined by the lack of a sub G1 peak in DNA histograms (Figure 1A,B). Several studies have shown the threshold to observe metformin-induced cell death to be approximately 48 h. To ensure that our conditions did not result in rapid cell death in the window prior to cell cycle analysis, we performed viability assays at the 48 h time point. Sytox Red cell toxicity assays demonstrated that metformin had a minimal effect at 48 h, resulting in decreased viability from approximately 95% (100 μM metformin, control) to approximately 86% at 10 mM metformin (Figure 1C). These findings confirm that metformin’s capacity to induce cell death is largely dependent on concentration, and that concentrations below 1 mM failed to affect proliferation or cancer cell viability. 

### 2.2. Mitochondrial Superoxide Production Is Attenuated by Metformin

**Figure 2:**
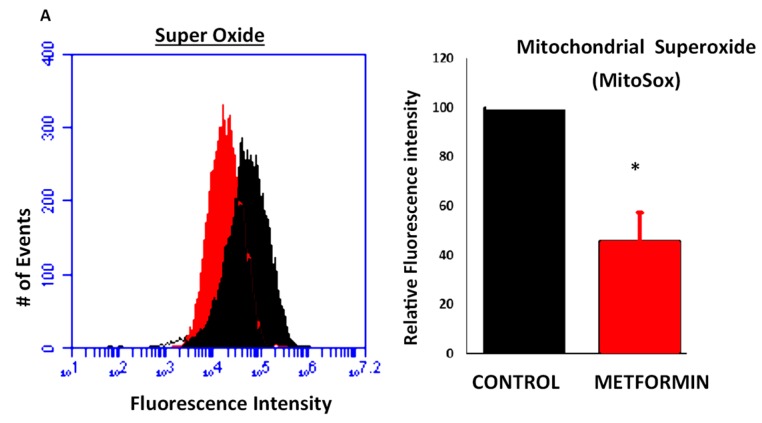
Metformin alleviates endogenous ROS production and subsequent pJNK activation. MDA-MB-231 cells cultured in the presence (red) or absence (black) of metformin (100 µM) for 48 h and subsequently stained with MitoSOX mitochondrial superoxide detection indicator. Fluorescence intensity was ascertained by flow cytometry and was normalized to untreated control. Studies were performed in triplicate, with 10,000 events each, and compared using Student’s *t*-test, * *p* ≤ 0.05.

Oxidative stress is associated with carcinoma-stromal co-evolution, and is also associated with inflammatory signaling in cancer [17]. Metformin has been shown to repress the production of ROS, though some studies have also suggested that metformin itself is an inducer of oxidative stress [18]. As such, we sought to determine whether metformin could attenuate endogenous ROS production and inhibit inflammatory signaling in MDA-MB-231 cells using the fluorogenic MitoSOX ROS detection system with flow cytometric detection. MDA-MB-231 cells were incubated with metformin for 48 h, and subsequent ROS detection was performed via flow cytometry where increased mean fluorescence intensity is associated with greater oxidative stress. In metformin-exposed cells, the measurement of superoxide staining was reduced by 40% as compared to control (Figure 2). These findings support a model in which metformin reduces cellular oxidative stress in cancer cells, resulting in decreased stress signaling.

### 2.3. Metformin Inhibits Expression of Inflammatory Mediators COX2 and ICAM1 in MDA-MB-231 Cells

**Figure 3:**
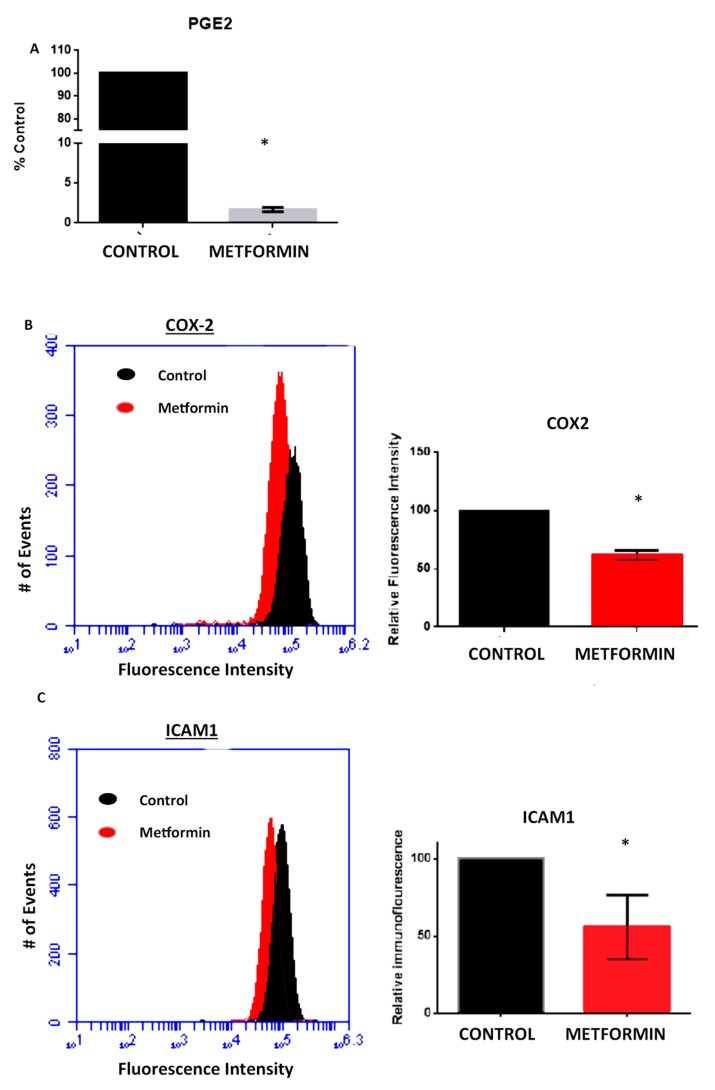
Metformin represses expression of pro-inflammatory markers in breast cancer. (**A**) MDA-MB-231 cells were incubated with or without metformin for 3 days and levels of PGE2 in the culture supernatant measured by competitive ELISA. MDA-MB-231 breast cancer cells were cultured in the presence or absence of metformin for 48 h after which cells were fixed and immunofluorescently stained for (**B**) COX2 or (**C**) ICAM1 protein expression. Staining intensity was measured by flow cytometry and normalized to control for comparison (right of histogram). Flow cytometry assays were performed in quadruplicate with 10,000 events registered per replicate. ELISA was performed with 4 technical repeats on 2 experiments. Significance was determined using Student’s *t*-test, where * *p* ≤ 0.05.

ROS has been directly correlated with the expression of inflammatory signaling molecules such as COX2. Interestingly, inflammatory signaling has also been shown to be repressed by metformin [19]. Since COX2 is a central mediator in the inflammation/cancer signaling axis and has been associated with increased tumor grade and poorer prognosis among patients with estrogen-independent breast cancer [20,21], we were interested in ascertaining the impact of metformin on COX2 activity and expression. Competitive ELISA assays were conducted with PGE2 (the enzymatic product of COX2) and results showed that metformin drastically repressed PGE2 levels in the supernatant of MDA-MB-231 cells after a 72-h incubation with metformin (Figure 3A). Additionally, we observed that after 48-h incubation in the presence or absence of metformin, COX2 expression was suppressed by approximately 30%, suggesting that metformin indeed elicited its effects in part due to repression of COX2 (Figure 3B). 

In a separate study, we found that metformin greatly reduced nemosis-induced ICAM1 expression in primary human dermal fibroblasts (Figure S1). ICAM1, a cell surface protein which is directly involved in cellular transmigration, has been reported to be induced by ROS and is associated with increased invasiveness and metastasis of breast cancer cells [22,23,24]. As such, we investigated the ability of metformin to alter the expression of ICAM1 in breast cancer cells using immunofluorescence and flow cytometry. After a 48-h incubation, metformin repressed expression of ICAM1 by 40% of control (Figure 3C). As ICAM1 is directly associated with cell migration, this provides a mechanistic link between metformin and abrogation of cancer cell invasiveness.

### 2.4. Metformin Inhibits in Vitro Migration, Invasion, and Ex Ovo Metastasis of MDA-MB-231 Cells

**Figure 4:**
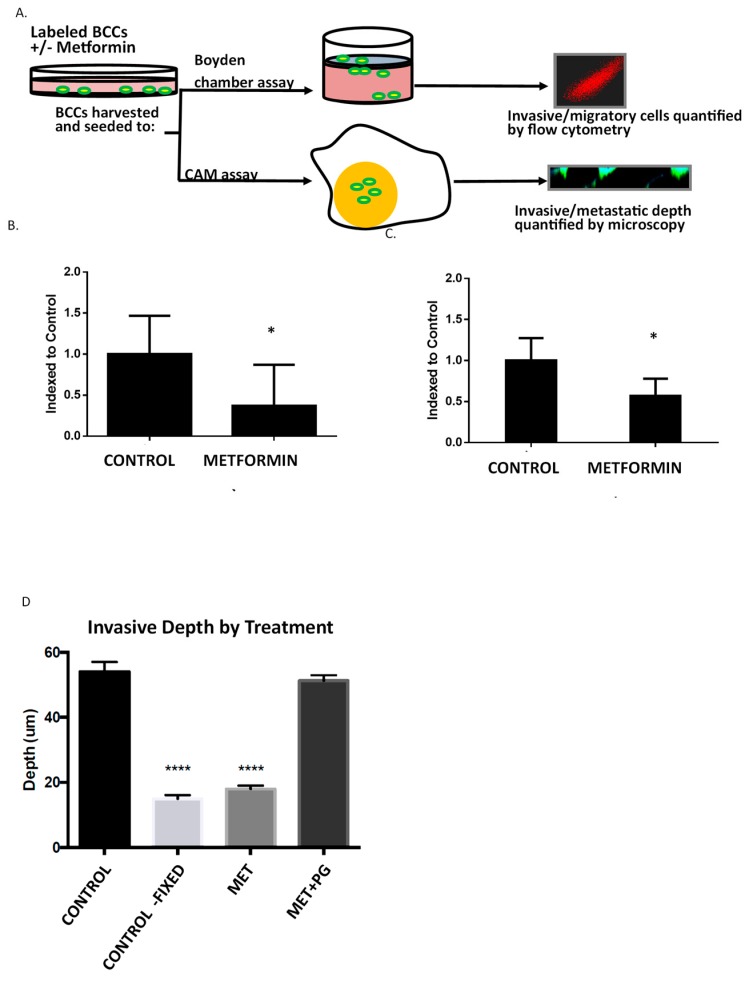
Metformin attenuates breast cancer cell migration, invasion, and metastasis. (**A**) MDA-MB-231 cells were pre-exposed to metformin for 48 h, collected, and stained with CellTracker Green fluorescent stain. Stained cells were ceded in the upper chamber of a Boyden chamber plate in the absence (**B**), or the presence (**C**) of Matrigel coating. The number of transmigratory/invading cells in response to chemoattractant (DMEM with 10% FBS) were enumerated by flow cytometry and normalized to control. (**D**) Anti-metastatic action of metformin is reversed by PGE2. MDA-MB-231 cells were labeled with CellTracker Green (10 μM) and seeded onto mesh squares placed on the chorioallantoic membrane (CAM) of 10-day old chicken embryos grown ex ovo. After 3 days, portions of the CAM-containing human cells were removed, fixed, stained with DAPI and visualized by fluorescence microscopy using an inverted laser confocal microscope with *Z*-axis control. Images were taken every 1 μm for approximately 100 μm, and the invasion maximum was defined by the leading edge of fluorescent cell invasion. Significance was determined by ANOVA and Tukey’s post hoc analysis, **** *p* ≤ 0.01, * *p* ≤ 0.05.

Given that proliferation was largely unaffected at pharmacologically relevant concentrations of metformin, despite the suppression of COX2 and ICAM1 expression, we investigated the impact of low dose metformin on cell migration and invasiveness using Boyden Chamber Flow Cytometry (BCFC) (Figure 4A). Briefly, MDA-MB-231 cells were incubated in the presence or absence of 100 μM metformin for 48 h (Figure 4A, upper) [25]. CMFDA (5-chloromethylfluorescein diacetate)-loaded MDA-MB-231 cells were seeded in the upper well of a Boyden migration or invasion chambers with 10% fetal bovine serum used as a chemoattractant in the lower chamber. After overnight incubation, fluorescent transmigratory cells were enzymatically detached and the number of fluorescent cells determined using flow cytometry. Cell migration (in the absence of extracellular matrix) was repressed by approximately 63% (Figure 4A). In the presence of extracellular matrix, invasion was repressed by approximately 40% (Figure 4B). Together, these findings support the contention that low dose metformin plays a role in repressing key features of breast cancer metastasis, which may in turn contribute to its proposed beneficial effect in breast cancer therapies.

To determine if changes in cell migration observed in BCFC studies would translate to a more physiologically relevant system, we employed chorioallantoic membrane (CAM) assays as models of metastasis as described previously (Figure 4A, lower) [26]. Briefly, MDA-MB-231 cells were incubated in the presence or absence of metformin for 48 hrs. Cells were harvested and fluorescently stained with CMFDA, and grafted to chick embryos. After 72 h, chick embryos were analyzed by confocal microscopy to determine the number and depth of tumor cell metastasis from the original graft. MDA-MB-231 cells overexpress COX2, which is largely responsible for PGE_2_ production in the tumor microenvironment. As such, we also investigated the ability of PGE_2_ to antagonize metformin’s effect on cell migration by concomitant administration of 100 nM PGE_2_ during metformin incubation. Here, we observed that metformin pretreatment caused a complete abrogation of cell metastasis in CAM-metastasis assay as compared to control CAMs (Figure 4D). Furthermore, PGE2 reversed this effect, suggesting that metformin’s anti-metastatic activity is dependent on its ability to repress PGE2 production and subsequent pro-invasive inflammatory signaling.

### 2.5. COX2 Expression is Correlated With Decreased DMFS in TNBC

**Figure 5:**
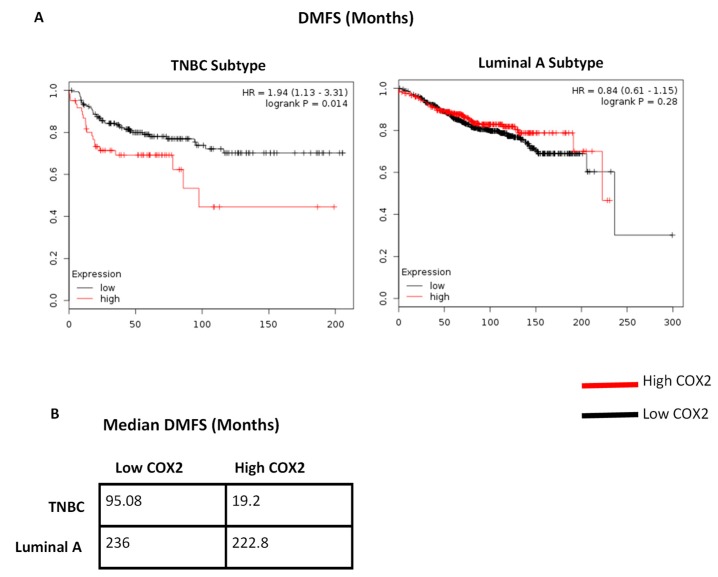
COX2 expression has prognostic significance in basal, but not Luminal A intrinsic breast carcinoma subtypes. (**A**) Interrogation of public microarray repositories using KMPLOT online analysis tool (KMPLOT.COM), DMFS was compared between those with high COX2 expression to those with low COX2 expression, in patients with either basal or luminal breast cancer subtypes. The median time of DMFS in the same cohort is summarized in (**B**).

There has not been a clear consensus on the prognostic significance of COX2 expression in breast cancer. Recently, however, it was reported that in a small cohort of patients with high grade triple negative breast carcinoma, immunohistochemical COX2 expression was associated with decreased 5-year disease-free survival [20]. Using the KMPLOT online analysis tool (http://kmplot.com/analysis/index.php?p=service&cancer=breast), we compared the significance of COX2 expression levels in TNBC versus luminal A breast cancer subtypes. We found that high COX2 expression (above median) in basal breast cancer is directly correlated with decreased distant metastasis free survival (DMFS) as compared to those patients with low COX2 expression (19.2 months vs 95.08 months respectively) (Figure 5A,B). No such correlation was observed among patients with luminal breast cancer whose median DMFS was 236 months for those with low COX2 expression and 222.8 months for patients with high COX2 expression. These studies support the notion that metformin’s ability to repress COX2 in breast carcinoma could potentially be central to its chemopreventive properties, but that effect might be limited to patients with basal-like breast cancer subtypes.

## 3. Discussion

Epidemiological evidence suggests that metformin usage is associated with significantly better patient outcomes among diabetic patients with cancer. However, much controversy has surrounded the putative mechanism by which that might occur. There is little dispute that metformin is capable of inducing apoptosis and cell cycle arrest in experimental breast cancer models; multiple in vitro studies including our own have demonstrated this effect in MDA-MB-231, MDA-MB-468, MCF-7, SKBR3, as well as other cell lines [13,14,27]. However, in nearly every case, concentrations of metformin were above 1 mM, and there are some questions as to the achievability of that concentration in the clinical setting. A recent pharmacokinetic study focusing on this issue showed that metformin, when administered at 350 mg/kg by intraperitoneal injection in mice, resulted in an average intratumoral concentration of 77 μM in HCT116-derived xenograft tumors [10]. This suggests that the concentrations of metformin (1–20 mM) that have typically been associated with proliferative arrest or apoptosis are not likely to be reflected in vivo at doses which are clinically tolerable, and that an alternative mechanism the drugs antineoplastic mechanism may be likely. Supporting this contention, a 2013 study showed that tumor biopsies from metformin treated patients trended toward a decrease in proliferation, not apoptosis, as indicated by Ki67 and TUNEL staining, respectively [2]. These findings support a model in which metformin does not directly impact cancer cell survival but may have other cell non-autonomous effects to impact neoplastic proliferation and progression.

**Figure 6:**
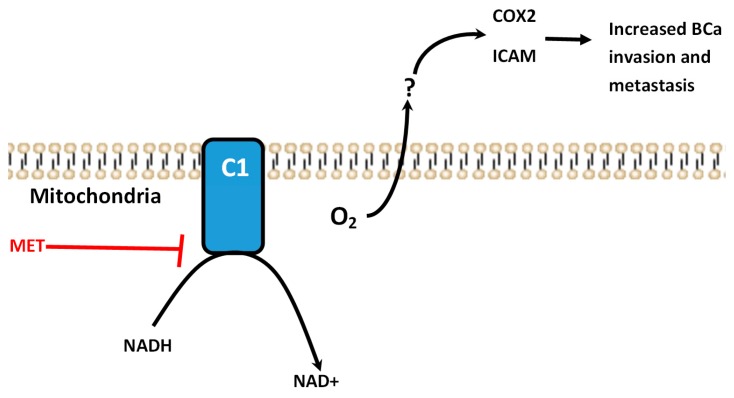
Model for metformin anti-neoplastic activity. In this model, superoxide is generated in cancer cells during mitochondrial respiration as a by-product of NADH reduction in cancer cells. ROS activity leads to COX2 and ICAM1 expression, as well as induction of other inflammatory mediators, resulting in increased metastatic potential in tumor cells. Metformin disrupts the function of complex I, thereby preventing ROS generation and attenuating metastasis.

Pathologic ROS production in cancer has been attributed to mitochondrial dysfunction as well as overexpression of NADPH Oxidases (NOX) [17]. This increase is typically considered to be a key event in maintaining an inflammatory microenvironment, in part by supporting sustained activation of JNK/SAPK pathways [28]. As a result, COX2 expression and subsequent prostaglandin production are increased, thereby perpetuating a microenvironment which supports tumor cell invasiveness (Figure 6) [20]. Here we have shown that metformin reduced production of endogenous ROS and represses COX2 and ICAM1 expression (Figure 2 and Figure 3). The primary source of ROS production in our particular model is unclear. However, metformin has been shown to repress ROS production via NOX in pancreatic cancer cells [29]. The loss of ROS production in MDA-MB-231 cells resulted in the commensurate loss of COX2/prostaglandin E2 dependent cell migration (Figure 4, Figure 1). Interestingly, other studies have suggested the metformin induces ROS production at higher concentrations, further supporting our assertion that metformin functions through distinct mechanisms at lower vs higher concentrations [15]. A recent study, however, showed that at mM concentrations metformin can repress oxidative stress [18]. These studies provide a different lens through which to view the antineoplastic effects of metformin and may provide insight into other therapeutic applications for metformin where endogenous ROS production and autocrine inflammatory signaling may play a role.

These studies suggest that metformin’s role in the management of cancer may not function as a type of cytotoxic therapy, but may function more as a chemopreventive agent which repress the metastatic and invasive properties of cancer. As such, this would alter the therapeutic context in which metformin should be studied in the clinical setting. It might be more effective to implement metformin to prevent tumor recurrence in an adjuvant setting or for the treatment of patients with documented high risk for breast cancer. 

## 4. Materials and Methods

### 4.1. Tissue Culture

MDA-MB-231 cells were extracted from human breast adenocarcinoma (metastatic) and acquired from the American Type Culture Collection (ATCC). Cells were maintained in Dulbecco’s modified eagle medium (DMEM) supplemented with 10% fetal bovine serum (FBS) and 1% penicillin/streptomycin antibiotic/antimycotic. Cultures were incubated at 37 °C and 5% CO_2_ and sub-cultured twice weekly at a 1:4 ratio.

### 4.2. Migration and Invasion

MDA-MB-231 cells were plated in 100 mm culture dishes at a density of 3 × 10^6^ cells per plate. After 24 h, cells were cultured with low-glucose (1 g/dL) DMEM (LG-DMEM) in the absence (control) or presence of 100 μM metformin for 48 h under incubation (37 °C). Cells were trypsinized, subsequently labelled with CellTracker™ Green (Thermo Fisher Scientific, Waltham, MA, USA) and incubated at 37 °C for 30 minutes. The cells were then transferred at a density of 4 × 10^4^ cells per well in the upper chamber of a 24-well Boyden Chamber filtration plate (migration assay) or ECMatrix (invasion assay) plate. The bottom wells contained 10% FBS DMEM as a chemoattractant or serum-free DMEM (negative control). Cells were incubated for 24 h and cells from the lower membrane and lower chamber were collected by trypsin dissociation. Labeled fluorescent transmigratory cells were enumerated using flow cytometry after gating, and represented as % control. All experiments were conducted at least 3 times with 6 technical repeats.

### 4.3. CAM Metastasis Assays 

Fertilized white leghorn chick embryos were obtained from Charles River Laboratories (Wilmington, MA, USA). Following 2 days of incubation in a 37 °C humidified incubator, eggs were swabbed with 70% ethanol and embryos were carefully removed from the shell using a Dremel tool and placed in a sterile, covered weigh boat and returned to the incubator. On day 10 of incubation, 5 × 10^5^ MDA-MB-231 cells were labeled with 10 μM CellTracker Green CMFDA (Thermo Fisher Scientific, Waltham, MA, USA), re-suspended in 100 μL PBS, and added onto the CAM on a nylon mesh for localization of the cells as previously described [26,30,31,32,33]. After 3 days of incubation, the areas of interest were extracted, fixed in 4% paraformaldehyde and mounted with ProLong Gold Anti-fade Mountant with DAPI (Thermo Fisher Scientific, Waltham, MA, USA). Depth of invasion from the CAM surface was defined as the leading edge of invading cells in randomly selected fields. Images were acquired on Nikon laser confocal microscope (Melville, NY, USA) using Nikon elements software. Six onplants were set to each CAM, with 5 CAMs per experimental group.

### 4.4. Reactive Oxygen Species (ROS)

MDA-MB-231 cells were plated in 12-well plates at a density of 1 × 10^5^ cells per well. Cells were cultured in LG-DMEM, in the presence or absence of metformin for 48 h. Cells were trypsinized, pelleted, and incubated in MitoSOX^®^ Red (Thermo Fisher Scientific) staining solution at 37 °C for 15 minutes. Fluorescence intensity as a measure of oxidative stress was determined by flow cytometry. Measurements were indexed to control. A minimum of 10,000 events were collected and the experiment repeated 4 times. 

### 4.5. PGE_2_ ELISA

PGE2 ELISA competitive assay was performed in accordance with the manufacturer’s instructions with some alterations (Cayman Chemical PGE2 monoclonal Kit, Cayman Chemical, Ann Arbor, MI, USA). MDA-MB-231 cells were cultured for 3 days in the presence or absence of metformin as described above. 50 μL of culture media was added to each well of the provided anti-mouse IgG coated 96-well plate in quadruplicate, followed by addition of PGE2-Acetylcholinesterase conjugate tracer and PGE2 monoclonal antibody. After overnight incubation, wells were washed and developed with Ellman’s reagent for 90 minutes. The plate was read using a BioRad model 550 plate reader at an absorbance of 415 nm. For each sample, the binding ratio to maximum binding (B/B0) was determined. B/B0 values were subsequently fitted to a standard curve and analyzed to determine percent control of PGE2 production. The experimental was repeated twice with 4 technical replicates. 

### 4.6. Immunostaining

Immunostaining for detection of ICAM1 and COX2 were performed as described previously [34]. Briefly, for flow cytometry assays, 1 × 10^6^ MDA-MB-231 cells which had been incubated in LG-DMEM in the presence or absence of 100 µM metformin for 72 h were detached, fixed, and immunostained with α-ICAM1 (Thermo Fisher Scientific, Waltham, MA, USA) or α-COX-2 (Bethyl Laboratories, Montgomery, TX, USA) primary antibodies, followed by secondary staining with Alexa 647 goat α-rabbit antibodies (Thermo Fisher Scientific, Waltham, MA, MA). Fluorescence intensity was measured by flow cytometry. For flow cytometry, a minimum of 3 repeats were performed, collecting at least 10,000 events each. 

### 4.7. Cell Cycle Analysis

After 72 h in the presence or absence of increasing concentrations of metformin, MDA-MB-231 cells were trypsinized and fixed in ice-cold 70% ethanol for 30 minutes at 4 °C. Cells were washed and re-suspended in phosphate buffered saline (PBS) with 5 µL of RNase. Propidium iodide was added to each sample of cells and samples were analyzed by flow cytometry. Forward and side scatter parameters were used to exclude cellular debris. Sub-G1/G0, G1/G0, S and G2/M phases were identified and compared across exposure groups. Studies were performed in quadruplicate.

### 4.8. Data Mining

Data mining for Kaplan-Meier survival was performed as described previously [34,35]. Briefly, Kaplan-Meier survival analysis was performed utilizing KM plotter server (kmplot.com), which analyzes breast cancer patient survival data from public microarray data repositories GEO (Gene Expression Omnibus, https://www.ncbi.nlm.nih.gov/geo/), TCGA (The Cancer Genome Atlas, https://cancergenome.nih.gov/), and EGA (European Genome-phenome Atlas, https://www.ebi.ac.uk/ega/). Patients were stratified as COX2-low or COX2-high according to the median expression values for COX2 throughout the cohort of patients with basal-like (879 patients) or luminal intrinsic subtypes (2504 patients) of breast carcinoma in accordance with Affymatrix probe 204748_at.

### 4.9. Statistical Analysis

Significance was determined for migration, invasion, PGE2 ELISA, cell cycle, and CAM metastasis assays using 2-way ANOVA followed by Fisher’s LSD for post hoc analysis to compare treatment groups to control. For flow cytometric immunostaining and CellROX assays, significance was determined using Student’s *t*-test. Prism GraphPad (Version 6.04, GraphPad Software, La Jolla, CA, USA) was used for all analyses. Significance was determined where *p* ≤ 0.05.

## 5. Conclusions

In conclusion, we have shown that exposing invasive TNBCs with sub-apoptotic concentrations of metformin results in significant reductions in both invasion and metastasis, and additionally reduce expression of inflammatory mediators COX2, JNK and ICAM1. Taken together with metformin’s inhibition in ROS production, these studies demonstrate that at pharmacologically achievable concentration, metformin may contribute to improved metastasis free survival in TNBC patients.

## Supplementary Materials

Supplementary materials can be found at http://www.mdpi.com/1422-0067/19/11/3692/s1.

## Abbreviations

TNBC	triple negative breast cancer
ROS	reactive oxygen species
BCFA	Boyden Chamber-Flow assay
CAM	Chorioallantoic assay
ELISA	enzyme-linked immunosorbent assay
COX2	cyclooxygenase 2
ICAM1	intercellular adhesion molecule 1
PGE_2_	prostaglandin E2
IGF-1	insulin-like growth factor 1
CMFDA	5-chloromethylfluorescein diacetate
DMFS	distant metastasis free survival
DMEM	Dulbecco’s modified eagle medium
FBS	fetal bovine serum
ATCC	American Type Culture Collection
NOX	NADPH oxidases
