# Supplementary material for: Metformin Inhibits Migration and Invasion by Suppressing ROS Production and COX2 Expression in MDA-MB-231 Breast Cancer Cells
Source: Int J Mol Sci. 2018 Nov 21;19(11):3692. doi: 10.3390/ijms19113692 (PMC6274682; doi:10.3390/ijms19113692)
Supplement: Supplementary file 1 [file ijms-19-03692-s001.pdf]

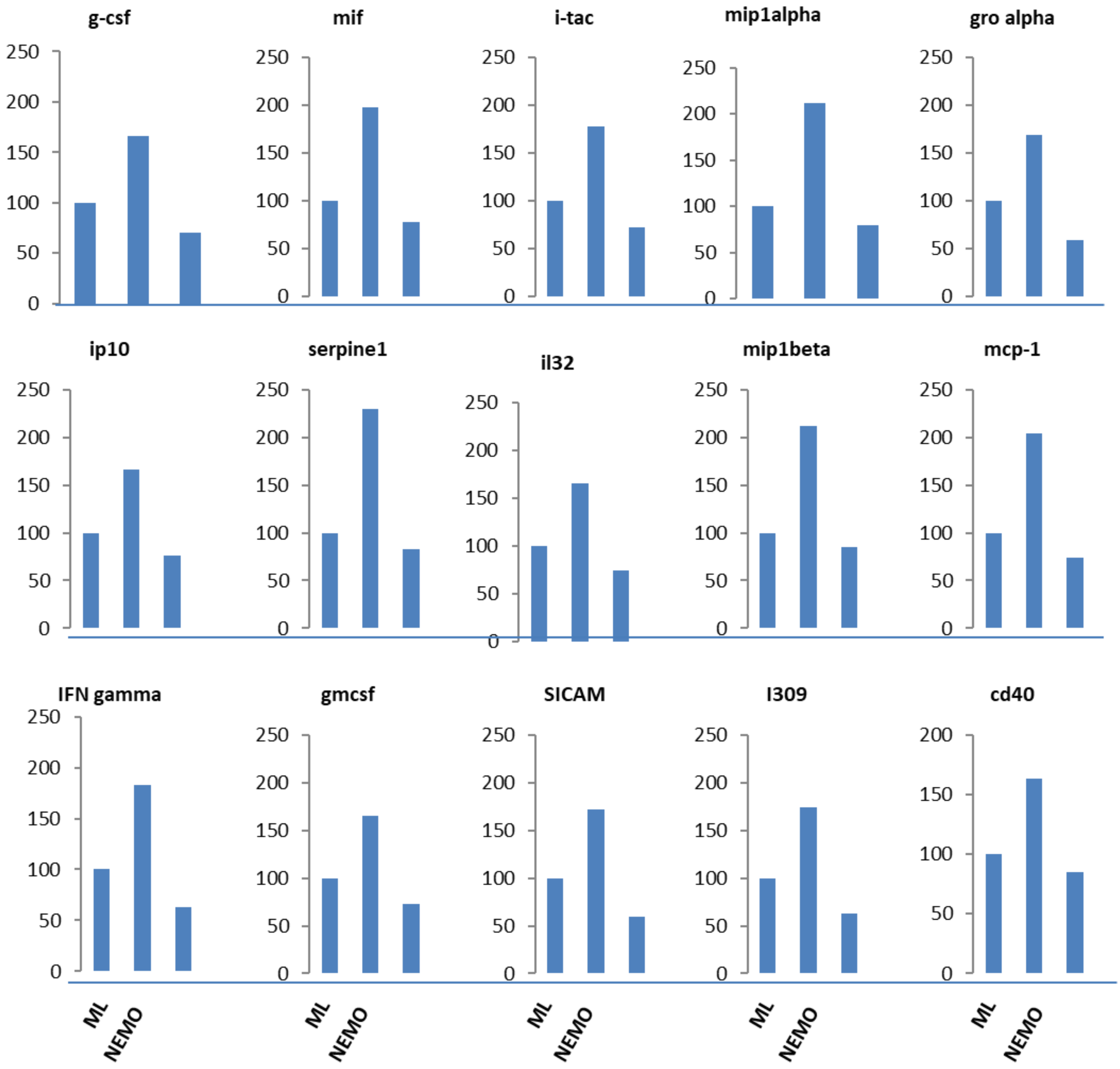

Supplement 1. Metformin reduces nemosis-induced inflammatory responses in human derma fibroblasts (HDFn). HDF cells were cultured under monolayer or low attachment conditions in the presence or absence of100µM metformin. After 24 hours, the supernatants for the cultures were probed using a targeted antibody array (R&D Systems). Metformin attenuated the inflammatory response, as evidenced by blunting the expression of nemesis induced pro-inflammatory cytokines.
